# Cerebrospinal Fluid Markers in Sporadic Creutzfeldt-Jakob Disease

**DOI:** 10.3390/ijms12096281

**Published:** 2011-09-23

**Authors:** Gianluigi Zanusso, Michele Fiorini, Sergio Ferrari, Alberto Gajofatto, Annachiara Cagnin, Andrea Galassi, Silvia Richelli, Salvatore Monaco

**Affiliations:** 1 Section of Neuropathology, Department of Neurological, Neuropsychological, Morphological and Motor Sciences, University of Verona, Policlinico G.B. Rossi, Piazzale L.A. Scuro 10, 37134 Verona, Italy; E-Mails: michele.fiorini@univr.it (M.F.); sergio.ferrari@ospedaleuniverona.it (S.F.); 2 Section of Clinical Neurology, Department of Neurological, Neuropsychological, Morphological and Motor Sciences, University of Verona, Policlinico G.B. Rossi, Piazzale L.A. Scuro 10, 37134 Verona, Italy; E-Mails: alberto.gajofatto@univr.it (A.G.); silvia.richelli@univr.it (S.R.); 3 Section of Neurology, Department of Neurosciences, University of Padova, Via Giustiniani 5, 35128 Padova, Italy; E-Mail: annachiara.cagnin@unipd.it; 4 Section of Legal Medicine, Department of Health Care Direction, San Bortolo General Hospital, Via Rodolfi 37, 36100 Vicenza, Italy; E-Mail: autospia@yahoo.it

**Keywords:** sporadic Creutzfeldt-Jakob disease, 14-3-3 protein, tau protein, amyloid beta peptide

## Abstract

Sporadic Creutzfeldt-Jakob disease (sCJD) is the commonest form of human prion diseases, accounting for about 85% of all cases. Current criteria for *intra vitam* diagnosis include a distinct phenotype, periodic sharp and slow-wave complexes at electroencephalography (EEG), and a positive 14-3-3-protein assay in the cerebrospinal fluid (CSF). In sCJD, the disease phenotype may vary, depending upon the genotype at codon 129 of the prion protein gene (*PRNP*), a site of a common methionine/valine polymorphism, and two distinct conformers of the pathological prion protein. Based on the combination of these molecular determinants, six different sCJD subtypes are recognized, each with distinctive clinical and pathologic phenotypes. We analyzed CSF samples from 127 subjects with definite sCJD to assess the diagnostic value of 14-3-3 protein, total tau protein, phosphorylated_181_ tau, and amyloid beta (Aβ) peptide 1-42, either alone or in combination. While the 14-3-3 assay and tau protein levels were the most sensitive indicators of sCJD, the highest sensitivity, specificity and positive predictive value were obtained when all the above markers were combined. The latter approach also allowed a reliable differential diagnosis with other neurodegenerative dementias.

## 1. Introduction

Prion diseases, or transmissible spongiform encephalopathies (TSEs), are fatal neurodegenerative disorders of human and animals, occurring as sporadic, genetic and iatrogenic forms [1]. The crucial event in the pathogenesis of TSEs is the conformational conversion of the cellular prion protein, or PrP^C^, into PrP^Sc^, a self-propagating protease-resistant isoform. Distinct sites of endogenous or exogenous PrP^Sc^ proteolytic cleavage, generating a core fragment named PrP27-30, characterize different prion strains [2,3]. Sporadic Creutzfeldt-Jakob disease (sCJD), the most common human TSE, accounts for nearly 85% of all human prion diseases and has an annual incidence of 1–2 cases per million. After its recognition in 1922, different clinical subtypes have been described based on signs and symptoms at presentation, age at onset, survival time, and diagnostic test results. Clinical forms of sCJD include the myoclonic or ‘classic’ sCJD, the Heidenhain’s variant, the cerebellar or ataxic Oppenheimer-Brownell variant, the thalamic and panencephalopathic subtypes. Over the last two decades, it has been assessed that the remarkable heterogeneity of sCJD phenotypes is influenced by the conformation of PrP^Sc^ (type 1 or type 2 PrP27-30) and by the polymorphic codon 129 of the prion protein gene (*PRNP*), a site of methionine (M)/valine (V) polymorphism. Currently, a sextet of molecular subtypes of sCJD are recognized, named MM1, MV1, VV1, MM2, MV2 and VV2 [4].

A definite diagnosis of sCJD is obtained following the demonstration of the pathologic prion isoform in brain tissues; possible or probable sCJD is defined on the basis of clinical features, periodic sharp and slow waves at electroencephalography (EEG), altered signal at brain MRI, and a positive 14-3-3 protein assay in the cerebrospinal fluid (CSF) [5,6].

A number of studies devoted to CSF analysis in sCJD and other neurodegenerative conditions have assessed the diagnostic sensitivity and specificity of different surrogate markers indicative of neuronal degeneration or glial cell activation, including 14-3-3 protein, tau protein, phosphorylated tau, S100 protein, neuronal specific enolase, and amyloid-β (Aβ)_1-42_, either alone or in combination [7–11]. The best investigated CSF markers, namely 14-3-3 proteins, are a group of cytosolic polypeptides, which are released in the CSF in disorders other than sCJD, encompassing stroke, infections, inflammatory events, epileptic seizures, and toxic-metabolic conditions [12–14]. Despite this apparent lack of specificity, it has been shown that within the appropriate clinical setting, a positive 14-3-3 assay may provide a diagnostic sensitivity and specificity of nearly 95–98% in sCJD [12,15]. The usefulness of the 14-3-3 assay is particularly evident in the differential diagnosis of rapidly progressive dementias (RPD) [16,17], especially when combined with quantification of tau protein, a microtubule-binding polypeptide, highly expressed in neurons and their axonal extensions [18–20].

Aim of the present study was to determine the diagnostic value of “neurodegeneration CSF markers” in sCJD. Given the wide phenotypic variability encountered in distinct sCJD subtypes, we retrospectively assessed the diagnostic value of CSF markers, either within distinct molecular subtypes or in comparison to other dementias. Therefore, we evaluated 14-3-3 protein as well as CSF levels of tau, phosphotau_181_ and Aβ_1-42_ in 127 cases of definite sCJD, 5 subjects with familial CJD (fCJD), 7 patients clinically diagnosed with Alzheimer’s disease (AD), and 15 patients with frontotemporal dementia (FTD). We assessed the utility and the diagnostic value of each single marker as well as the combination of different markers.

## 2. Materials and Methods

### 2.1. CSF and Patients

We studied CSF samples from 127 subjects with definite sCJD cases and 5 patients with fCJD (3 subjects carrying E200K and 2 V210I mutations). Samples were collected during the period January 1999 through December 2010, from individual undergoing lumbar puncture for diagnostic purposes. A clinical history of the current and past illnesses was obtained, as well as results of EEG and brain MRI. Control groups consisted of 7 subjects with AD, 15 with FTD, 4 with herpes encephalitis, and 15 non-neurological patients. The study was done in accordance with the current revision of Declaration of Helsinki and the Good Clinical Practice guidelines. A written consent for all diagnostic procedures, including genetic studies, was obtained.

### 2.2. PRNP Codon 129 Genotype, PrP^Sc^ Typing and Molecular Classification

Search of *PRNP* mutations and M/V polymorphism at codon 129 were carried out on DNA extracted from blood specimens or from frozen brain tissues, according to standard methods. PrP^Sc^ typing was determined on brain homogenates obtained from different areas, after treatment with proteinase-K for 1 h at 37 °C. The immunoblot profile of PrP^sc^ was classified as type 1 or type 2, as previously described [3]. Each sCJD case was classified according to the genotype (M or V) at codon 129, the PrP^Sc^ type, and the neuropathological phenotype: 76 subjects were MM1, 7 MV1, 4 MM2, 20 MV2, and 20 VV2.

### 2.3. CSF Protein Analysis

CSF samples were stored at −80 °C prior to analysis. The 14-3-3 protein was assayed by immunoblotting, following SDS-PAGE. For this purpose, 10 μL of CSF was separated by SDS-PAGE and transferred to PVDF membranes (Immobilon P; Millipore) [14]. Each gel included 10 and 20 pg of recombinant 14-3-3 γ, CSF from definite sCJD cases, and normal controls. Membranes were incubated with anti-14-3-3 β polyclonal rabbit IgG cross-reacting with γ, ɛ, ζ, and η isoforms (Pan 14-3-3 antibody SC-629; Santa Cruz Biotechnology), at 1:500 dilution, then incubated with anti-rabbit immunoglobulin at 1:3,000 dilution. The reaction was revealed by an enhanced chemiluminescence system (ECL; Amersham). The presence of the 14-3-3 band was scored by two independent observers and classified as negative or positive based on comparison with positive controls and recombinant 14-3-3 protein. Total tau protein, phosphotau_181_ and Aβ_1-42_ were measured using enzyme immunoassays (Innogenetics, Ghent, Belgium). For total tau a threshold of 1300 pg/mL was selected as the cut-off value, with values higher than 1300 pg/mL being considered specific for sCJD [21].

### 2.4. Statistical Analyses

Statistical analyses were done using the program Excel (Microsoft). To compare continuous variables (presented as mean ± SD) between two groups, the unpaired samples t test was applied. The Chi-square test and the Fisher’s exact test were applied to categorical variables.

## 3. Results

### 3.1. Molecular, Demographic and Clinical Characteristics of sCJD Patients

MM1 subjects accounted for 59% of all sCJD cases and had mean disease duration of 6 months with a range of 2 weeks–17 months. In 90% of MM1 cases the clinical features were consistent with the ‘classic’ or Heidenhain’s phenotypes. Within the MV1 subtype, conventionally grouped with MM1 subjects, 4 out 7 patients had relatively long disease duration and an atypical clinical course. Conversely, MV2 and VV2 subtypes were similar as age at disease onset (67 ± 8 and 65 ± 8 years, respectively), but had different disease duration (MV2, 17.4 ± 10 and VV2, 7.4 ± 5.5 months; *p* < 0.002). Most VV2 patients displayed a subacute cerebellar syndrome rapidly complicated by dementia, while patients of the MV2 subtype, showed a more heterogeneous disease phenotype at presentation, with cerebellar and/or extrapyramidal/pyramidal signs, or cognitive impairment. The 4 subjects classified as MM2 cortical subtype (MM2C) had a younger age at onset (60 ± 4), a duration of 11 ± 9 months, and a clinical course characterized by behavioral changes/cognitive disturbances. The lag of time between disease onset and CSF analysis ranged from 2.5 ± 2.9 months in MM1 cases to 7 ± 5 in MV2 subjects. fCJD cases were younger at onset and had a disease phenotype mimicking ‘classic’ sCJD (Table 1).

### 3.2. CSF 14-3-3 Protein Assay and Tau Protein Levels

The 14-3-3 assay was positive in 96% of sCJD patients and in 100% of fCJD subjects. On the contrary, the 14-3-3 tested positively in 30% of AD and 13% of FTD. An upper cut-off threshold of 4000 pg/mL was selected for tau, since all reported values in non-sCJD are below this value. In 54% of sCJD subjects and in all fCJD cases, tau levels were over 4000 pg/mL. In contrast, in AD and FTD levels of tau protein were 898 ± 522 and 287 ± 194 pg/mL, respectively (Table 2). Statistical analyses showed that sCJD cases were significantly different from AD and FTD (AD *vs* sCJD *p* < 0.001; FTD *vs* sCJD *p* < 0.0001), but no differences were observed between AD and FTD. Therefore, in patients with dementia, in whom a differential diagnosis is required, 14-3-3 protein positivity and tau CSF levels over 1300 pg/mL tau represent reliable markers for sCJD.

### 3.3. CSF 14-3-3 Protein and Tau Protein Levels in Distinct sCJD Subtypes

As previously observed, the disease characteristics of different sCJD subtypes are highly variable, including cases with RPD and/or ataxic forms, and cases with slowly progressive cognitive decline and/or behavioral changes. Accordingly, we assessed 14-3-3 positivity and tau levels in each distinct subtype and we compared the results with other dementias. As expected, the 14-3-3 assay performed differently among different subtypes. While all MV1 and VV2 cases had a positive test, within the MM1 subtype, positivity was seen in 98.5% of the subjects, a value decreasing to 90% in MV2, and to 50% in MM2 subtype (Table 3). Among sCJD subtypes, variability was also observed for CSF tau levels (Table 3). Tau levels were below the 1300 pg/mL threshold in 2 MM1 subjects out of 76 (1 being also 14-3-3 negative), 1 MV1 case out of 7, and 5 MV2 patients out of 20 (one sample was 14-3-3 negative). The lowest frequency of 14-3-3 positivity (50%) was observed in the MM2C subtype, whereas 100% positivity was seen in the VV2 subtype. In 5 sCJD subjects, 3 MV2 and 2 MM2, who had tau levels below 1300 pg/mL at first CSF analysis, a second spinal tap was required for diagnosis (Table 4).

### 3.4. CSF Phosphotau_181_ and Aβ_1-42_ in sCJD Subtypes and Other Neurodegenerative Dementias

To investigate whether the CSF conventional diagnostic markers of neurodegenerative dementias might be helpful for sCJD diagnosis, we also evaluated phosphotau_181_ and Aβ_1-42_ levels in selected sCJD cases. In sCJD, despite the elevated CSF tau levels, the mean levels of phosphotau_181_ were 46 ± 22 pg/mL, and the ratio phosphotau_181_/tau was 0.014. On the contrary, phosphotau_181_ levels were 105 ± 47 pg/mL in AD (phosphotau_181_/tau, 0.17), and 37 ± 17 pg/mL in FTD (phosphotau_181_/tau, 0.16), values tenfold higher as compared to sCJD (Table 5).

**Figure 1:**
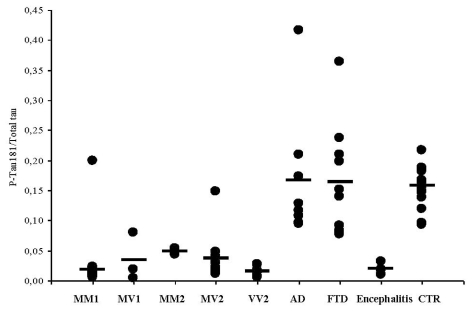
Phosphotau_181_/tau ratio in sCJD subtypes, FTD, AD, and herpes encephalitis.

**Figure 2:**
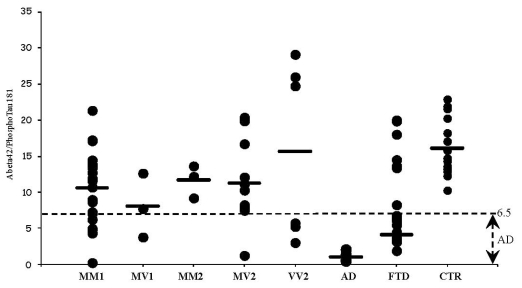
Aβ_1-42_/phosphotau_181_ ratio in sCJD subtypes, FTD, AD, and encephalitis.

Phosphotau_181_ values and phosphotau_181_/tau ratio did not significantly differ among distinct sCJD subtypes, whereas each subtype was different from AD (*p* < 0.001) and FTD (*p* < 0.002). Therefore, the use of CSF phosphotau_181_/tau ratio encourages its use as a sCJD marker (Figure 1). We next assessed the phosphotau_181_/tau ratio in the CSF of patients with herpetic encephalitis and we compared values with those seen in sCJD subjects. As expected, tau protein levels resulted elevated in subjects with encephalitis and the phosphotau_181_/tau ratio was low (0.019), thus overlapping data obtained in sCJD. These findings confirm that post-translation modifications of tau protein occur in AD and FTD, but not in sCJD. We also determined the levels of CSF Aβ_1-42_ in selected sCJD subjects of all available subtypes, AD and FTD. The rationale for doing so is the fact that Aβ1-42 is now included among the CSF markers supportive for AD diagnosis and staging. Interestingly, Aβ_1-42_ levels resulted significantly higher in all sCJD subtypes as compared to values obtained in AD and FTD. Additionally, we calculated the ratio Aβ_1-42_/phosphotau_181_ (Figure 2), a marker highly suggestive for AD diagnosis when values are below 6.5 [22]. In contrast to AD and FTD, the above ratio was higher than 6.5 in all sCJD subtypes, albeit in several subjects of all subtypes, except MM2, individual values were below 6.5.

## 4. Conclusions

The trend, in present-day searches for neurodegeneration biomarkers, is not just to find a single protein as an indicator of a distinct disorder, but rather to assess a panel of reliable biomarkers, whose combined expression will tend towards 100% sensitivity coupled with 100% specificity, a major goal in current research. To date, two main strategies have been pursued for the evaluation of CSF biomarkers in neurodegenerative disorders. These include the search for surrogate markers, to monitor the state and stage of disease on one hand, and the detection of proteins primarily involved in disease pathogenesis on the other hand. In sCJD, a positive CSF 14-3-3 assay is highly supportive of *intra vitam* diagnosis, provided there is an appropriate clinical setting, *i.e.* the presence of a typical EEG pattern and the occurrence of distinctive MRI changes. In sCJD patients included in our retrospective study, CSF 14-3-3 protein was positive in 96% of cases, while 100% of fCJD cases tested 14-3-3 positive. However, 30% of AD cases and 13% of FTD subjects also tested positive. These results are substantially in keeping with those reported in other investigations and further indicate that 14-3-3 is a highly sensitive marker with a low specificity profile. However, the absence of a standard protocol for 14-3-3 protein detection, whether qualitative or quantitative, may result in arbitrary determination, thus explaining the consistent variability among different studies [23,24]. Attempts to overcome this limitation have been provided by Gmitterova *et al*., who performed a quantitative ELISA analysis of 14-3-3 protein in distinct sCJD subtypes. In the foregoing study, the highest levels of 14-3-3 protein were observed in VV2, MM1 and MV1 subtypes, which are characterized by a rapid progression, while CSF 14-3-3 expression was significantly lower in MM2C and MV2 subtypes with long disease duration and atypical course [25]. Castellani *et al*. reached similar conclusions, confirming that the molecular subtype should be taken into account when interpreting results of the test [26].

**Figure 3:**
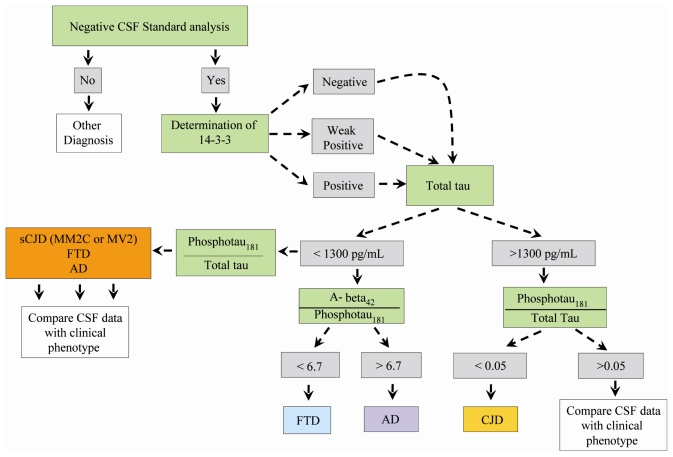
Chart for diagnosis of sCJD *versus* FTD and AD.

Our results on the assessment of tau levels across distinct molecular subtypes, show tau levels below the conventional threshold of 1300 pg/mL in a high proportion of MM2C and MV2 patients, in whom a repeated lumbar puncture was necessary. This confirms that in such cases, sCJD diagnosis might be less than simple and requires a follow-up of CSF markers as well as the determination of the codon 129. Different groups use arbitrary cut-off levels of tau, spanning from 1000 pg/mL to 1400 pg/mL. As shown in the present study, levels of tau protein higher than 1300 were found in sCJD only, with the exception of a single rapidly evolving AD case having tau levels of 1355 pg/mL. In contrast, in all subjects with FTD, tau levels were below the standard values [27]. The sensitivity and specificity of additional CSF markers, other than 14-3-3 protein and tau protein across sCJD subtypes, has never been previously assessed. Given the diagnostic difficulties encountered with MM2C and MV2 subtypes, we determined the diagnostic accuracy of Aβ_1-42_ or phosphotau_181_ in differentiating sCJD from other degenerative dementias. We found that the ratio phosphotau_181_/tau significantly differentiated all sCJD subtypes from AD and FTD. Therefore, we propose a diagnostic flow-chart which might be followed in a clinical setting of dementia in order to differentiate sCJD from non-sCJD forms, including AD and FTD (Figure 3).

## Figures and Tables

**Table 1:** Molecular and demographic data of sporadic Creutzfeldt-Jakob disease (sCJD) and familial CJD (fCJD) subjects.

Molecular subtype	Relative%	Gender F/M	Mean age (years)	Time from disease onset to lumbar puncture (months)	Disease duration (months)
MM-1 (*n* = 76)	59	33/43	70 ± 8 (53–99)	2.5 ± 2.9 (0.5–18)	6 ± 5 (0.5–27)
MV-1 (*n* = 7)	6	5/2	70 ± 7 (52–76)	4.1 ± 2.7 (1–9)	10.5 ± 4 (6–17)
MM-2 (*n* = 4)	3	2/2	60 ± 4 (54–62)	5.8 ± 3 (4–11)	11 ± 9 (4–18)
MV-2 (*n* = 20)	16	4/16	67 ± 8 (53–81)	7 ± 5 (2–24)	17.4 ± 10 (7–48)
VV-2 (*n* = 20)	16	11/9	65 ± 8 (54–79)	4.2 ± 3.2 (1–13)	7.3 ± 5.5 (3–25)
fCJD (*n* = 5)	-	3/2	57 ± 10 (47–73)	2 ± 1	5.8 ± 1.4 (4–8)

**Table 2:** Results of CSF 14-3-3 assay and tau protein levels in sCJD, fCJD, Alzheimer’s disease (AD), frontotemporal dementia (FTD), and controls.

Subjects	14-3-3 Protein positive	Tau protein levels (pg/mL) mean ± SD	Subjects with tau protein >4000 pg/mL
**sCJD** (*n* = 127)	122 (96%)	3305 ± 1096	79
**fCJD** (*n* = 5)	5 (100%)	4000	5

**FTD** (*n* = 15)	2 (13%)	287 ± 194	-

**AD** (*n* = 7)	2 (28%)	898 ± 522	-

**CTR** (*n* = 15)	-	188 ± 46	-

**Table 3:** 14-3-3 assay and levels of tau protein in sCJD subtypes and fCJD *vs* other dementias.

Molecular type	14-3-3 Positivity	Tau protein > 4000 pg/mL	Tau protein 1300< >4000	Tau protein < 1300 pg/mL
**MM-1** (n = 76)	75 (98.5%)	59 (78%)	15 (20%) (Mean: 2546 ± 870) (Range: 1443–3864)	2 (2.6%) (230,1272)
**MV-1** (n = 7)	7 (100%)	2 (28%)	4 (57%) (Mean: 2270 ± 1120) (Range: 1478–3062)	1 (14%) (650)
**MM-2** (n = 4)	2 (50%)	1 (25%)	1 (25%)	2 (50%) (609,445)
**MV-2** (n = 20)	18 (90%)	4 (20%)	11 (55%) (Mean: 2211 ± 1068) (Range: 1628–3792)	5 (25%) (Mean 892 ± 264)
**VV-2** (n = 20)	20 (100%)	13 (65%)	7 (35%) (Mean: 3433 ± 818) (Range: 1628–3897)	-
**fCJD** (n = 5)	5 (100%)	5	-	-
**AD** (n = 7)	2 (30%)	-	1507,1663	529 ± 336
**FTD** (n = 15)	2 (13%)	-	-	287 ± 194
**CTR** (n = 15)	-	-	-	188 ± 46

**Table 4:** sCJD patients with tau levels below 1300 pg/mL at first spinal tap.

Molecular subtype	First spinal tap		Lag between spinal taps (months)	Second spinal tap	
	14-3-3 Protein	Tau protein (pg/mL)		14-3-3 Protein	Tau protein (pg/mL)
MM-2	+/−	609	2	**+**	767
MM-2	+	445	4	**+**	2560
MV-2	+	507	15	**+**	3792
MV-2	+	1040	3	**+**	1925
MV-2	+	676	1	**+**	2659

**Table 5:** Cerebrospinal fluid (CSF) levels of tau and phosphotau_181_ in subjects with sCJD, AD, FTD and encephalitis.

Subjects	Tau protein pg/mL mean ± SD	PhosphoTau _181_ pg/mL mean ± SD	PhosphoTau _181_/Tau
**All sCJD** (*n* = 127)	3305 ± 1096	46 ± 22	0.014
**MM-1** (*n* = 76)	3456 ± 972	42 ± 17	0.019
**MV-1** (*n* = 7)	2772 ± 1394	48 ± 27	0.034
**MM-2** (*n* = 4)	1455 ± 1701	28 ± 4.4	0.049
**MV-2** (*n* = 20)	2211 ± 1068	53 ± 17	0.038
**VV-2** (*n* = 20)	3433 ± 817	56 ± 40	0.016
**AD** (*n* = 7)	898 ± 522	105 ± 47	0.17
**FTD** (*n* = 15)	287 ± 194	37 ± 17	0.16
**CTR** (*n* = 15)	188 ± 46	29 ± 8	0.16
**Encephalitis** (n = 6)	1814 ± 163	35 ± 11	0.019
